# Cultural and Personality Predictors of Facebook Intrusion: A Cross-Cultural Study

**DOI:** 10.3389/fpsyg.2016.01895

**Published:** 2016-12-02

**Authors:** Agata Błachnio, Aneta Przepiorka, Martina Benvenuti, Davide Cannata, Adela M. Ciobanu, Emre Senol-Durak, Mithat Durak, Michail N. Giannakos, Elvis Mazzoni, Ilias O. Pappas, Camelia Popa, Gwendolyn Seidman, Shu Yu, Anise M. S. Wu, Menachem Ben-Ezra

**Affiliations:** ^1^The John Paul II Catholic University of LublinLublin, Poland; ^2^Department of Psychology, University of BolognaBologna, Italy; ^3^Department of Clinical Neuroscience - Psychiatry, University of Medicine and Pharmacy “C. Davila” BucharestBucharest, Romania; ^4^Department of Psychology, Abant Izzet Baysal UniversityBaysal, Turkey; ^5^Department of Computer and Information Science, Norwegian University of Science and TechnologyTrondheim, Norway; ^6^Romanian AcademyBucharest, Romania; ^7^International Center for Research and Education in Innovative and Creative Technologies (CINETic) - National University of Theatre and Film I.L. Caragiale (UNATC)Bucharest, Romania; ^8^Psychology Department, Albright CollegeReading, PA, USA; ^9^Department of Psychology, University of MacauMacau, China; ^10^School of Social Work, Ariel UniversityAriel, Israel

**Keywords:** Facebook intrusion, cultural factor, personality, cross-cultural comparison, social network

## Abstract

The increase in the number of users of social networking sites (SNS) has inspired intense efforts to determine intercultural differences between them. The main aim of the study was to investigate the cultural and personal predictors of Facebook intrusion. A total of 2628 Facebook users from eight countries took part in the study. The Facebook Intrusion Questionnaire, the Ten-Item Personality Inventory, and the Singelis Scale were used. We found that two variables related to Country were significantly related to Facebook intrusion: uniqueness (negatively) and low context (positively); of the personality variables, conscientiousness, and emotional stability were negatively related to the dependent variable of Facebook intrusion across different countries, which may indicate the universal pattern of Facebook intrusion. The results of the study will contribute to the international debate on the phenomenon of SNS.

## Introduction

Social networking sites (SNSs) have become an important means of communication used for professional, private, recreational, and information purposes. At the same time, their rapid growth and wide popularity have spurred scholarly interest in their social impacts (Boyd and Ellison, 2007; Giannakos et al., 2013). The emergence of new communication tools—in this case, SNS—made it possible to make greater use of the Internet to establish interaction. The first SNS was launched in 1997 and currently there are hundreds of them worldwide. Apart from Twitter, LinkedIn, and MySpace, Facebook is one of the most popular ones. It was launched in 2004 and currently has about 1.79 billion monthly active users, giving them the opportunity to communicate and share information. Moreover, more than 85% of users are from outside of U.S. and Canada^1^. The increase in the number of SNS users is a global phenomenon (Vasalou et al., 2010). Despite the numerous “success stories” of online networks and communities (e.g., Boyd and Ellison, 2007), when Facebook was launched back in February 2004, few people would have predicted the magnitude of its success in the following years. The subject of Facebook is often taken up by researchers studying network communities due to its wide range of users and technological possibilities that allow us to maintain relationships from the offline world as well as establish new ones in the online world. Being a kind of platform for the exchange of information and a medium that makes it possible to establish or maintain existing relationships, Facebook has a huge impact on social life (Ellison et al., 2007; Mazzoni and Iannone, 2014). People aged from 13 to 35 constitute the highest percentage of users—these are young people, either entering, or about to enter the labor market (Pew Research Center, 2014). It can be assumed that for the youngest people Facebook will be one of the most common and natural forms of communication. All this makes the Facebook phenomenon an important issue and one that is worth researching. In addition, in previous studies there is a lack of a coherent model explaining the motivation to use Facebook, Facebook intrusion, and Facebook intensity (Błachnio et al., 2013).

Considering the increasing number of participants and the increasing amount of time spent using Facebook (Ryan et al., 2014), the problem of excessive usage is increasingly relevant and should be more profoundly investigated. Addiction to SNS can be treated as a subtype of Internet addition (Błachnio and Przepiorka, 2016). In the literature there is a term “Facebook intrusion,” which can be defined by Elphinston and Noller as excessive involvement in Facebook, disrupting day-to-day activities and interpersonal relationships (2011). The authors mention three aspects of this phenomenon, namely: (1) withdrawal, defined as a feeling of distress caused by the inability to access Facebook, (2) relapse and reinstatement, defined as unsuccessful efforts at reducing Facebook use, and (3) euphoria, defined as a feeling of connection with other people thanks to Facebook (Elphinston and Noller, 2011). Previous studies have shown that Facebook addiction is related to several psychological variables (Ryan et al., 2014). For example, subjective vitality and subjective happiness in a sample of Turkish students (Uysal et al., 2013; Satici and Uysal, 2015), life satisfaction, depression in a sample of Polish students (Błachnio et al., 2015) and lack of self-competence and well-being in a sample of Turkish students (Satici and Uysal, 2015) have been significantly associated with Facebook addiction. Facebook addiction was related to sleep disturbances, Extraversion, and Neuroticism were positively associated with Facebook addiction, whereas Conscientiousness was negatively related to it. Facebook intrusion was also related to dissatisfaction with relationships (Elphinston et al., 2011). Other results indicate that Facebook intrusion is also linked with chronotype (Blachnio et al., 2015). This list of possible psychological characteristics related to Facebook addiction reflects the complex relations among the variables. It should be pointed out that the list of possible predictors of Facebook addiction is not final and more studies focusing on psychological functioning of Facebook users are needed.

### The cultural aspect of facebook use

As regards the cultural aspect of Facebook, a review of the literature reveals that the previous studies only focused on comparing the patterns of SNS use in different countries (Singh et al., 2006; Gong et al., 2007). Vasalou et al. (2010) showed differences in Facebook use between different countries. For example, users from the UK spent more time on Facebook than users from other countries. For UK users, participation in Facebook groups was more important than for US users, while Italians preferred participating in groups, and playing games. In Greece, users regarded updating their profiles as the least important (Vasalou et al., 2010). Differences in the motivation to use Facebook were also revealed in the research carried out by Ji et al. (2010). Comparing three countries: South Korea, China, and the USA, they found that there were differences in the relations between loneliness, the sense of social capital, and SNS use. When communicating with other users, US users mainly focused on exchanging opinions and passing on information, while Korean and Chinese users were also concerned about the emotional climate of this form of communication (Ji et al., 2010).

The need of belonging is strongly related to cultural factors, especially to the individualism/collectivism dimension. In individualistic cultures (e.g., in the American culture), it is the individual's achievements and successes that are valued, while in collectivistic cultures (e.g., Chinese) the most important thing is harmony with the group and being part of it (Oyserman et al., 2002). Nadkarni and Hofmann (2012) hypothesized that people from individualistic cultures have a greater tendency to post private information and more often bring up controversial subjects on Facebook than users from collectivistic cultures. Jackson and Wang (2013) indicate that users from the Chinese culture, where collectivistic values dominate, use social networks less often than people from the American culture. A study among Palestinian youth in Abbas and Mesch (2015) indicated that cultural values such as collectivism, power distance, or uncertainty avoidance predict motivation for using Facebook for maintaining existing relationships.

So far, studies have drawn on Hofstede's theory (Hofstede, 1980), which describes culture on dimensions such as individualism—collectivism, uncertainty avoidance, power distance, or masculinity—femininity when attempting to determine intercultural differences in Facebook use (e.g., Ji et al., 2010). Power distance reflects the way people view the level of power distribution in their culture and accept their place in the social hierarchy. Individualism vs. Collectivism describes the level of integration with the group, which shows whether importance is placed on attaining personal or group goals. The Uncertainty—Avoidance dimension shows the degree to which societies tolerate unknown situations, changes, and unexpected events. The Masculinity vs. Femininity dimension reflects what kind of values are respected—for instance: assertiveness, ambition, or power are related to masculine values whereas human relationship building is characteristic for feminine values.

However, researchers point out that Hofstede's theory is difficult to use in research on motivation because of social, political, economic, and other changes experienced by various countries, and that this theory does not take the dynamics of these transformations into account. What is more, this theory does not successfully explain cultural differences in studies on the Internet. For this reason, in the present study we draw on the self-construal theory (Singelis, 1994). This theory concerning the independence and interdependence of the self and the other has been successfully used to predict media use (Tamam et al., 2009) and social media use (Kim et al., 2010).

Independent self-construal refers to concentration on being separate from others and on the desire to discover the unique proprieties of the individual, although the social group, and others are still important for people with independent self-construal. A crucial issue here is the autonomy and independence of the self and emphasis on setting and realizing individual goals. By contrast, for people with a high level of interdependent self-construal it is important to maintain interdependence and contact with others, which means perceiving themselves as inseparable from others. All decisions and behaviors of such persons are always analyzed with regard to others (Singelis, 1994). In a study of self-construal in 29 countries under Singelis' framework, there were four factors constituting this construct: group loyalty, understood as a sense of duty and obligation toward the group; uniqueness, defined as a feeling of uniqueness, and personal reward; low context, connected with directness in communication; and relational interdependence, understood as in-group harmony and relation (Fernández et al., 2005). Okabe (1983, p. 38; as cited in Abbas and Mesch, 2015, p. 8) characterized low context cultures as follows: “where very little is taken for granted, greater cultural diversity, and heterogeneity are likely to make verbal skills more necessary and, therefore, more highly prized.” The dimensions of low context and relational interdependence of the self are not related to individualism. Whereas, uniqueness and a low level of group loyalty are not related to masculinity culture, there is a preference in this culture for achievement and heroism, and the social roles are clearly distinct. Interdependent self-construal emphasizes social relationships and sharing—for example, sharing joy with others—while in independent self-construal it is autonomy and competition that are important (Fernández et al., 2005). Kim et al. (2010) suggested using the concept of self-construal for predicting behaviors connected with SNS. They indicated that people focused on social relationships and, consequently, characterized by strongly interdependent self-construal have higher motivation to strengthen relations through SNS and derive greater satisfaction from SNS use.

### Personality and facebook usage

There are many studies suggesting associations between Internet use and personality (Senol-Durak and Durak, 2011; Durak and Senol-Durak, 2014) and between Facebook use and personality characteristics (e.g., Moore and McElroy, 2012; Jenkins-Guarnieri et al., 2013; Seidman, 2013). According to Ryan and Xenos (2011), several studies have found that Facebook users are characterized by higher levels of extraversion (Wilson et al., 2010; Gosling et al., 2011; Ryan and Xenos, 2011) as well as by lower levels of conscientiousness (Wilson et al., 2010; Ryan and Xenos, 2011) and social isolation (Ryan and Xenos, 2011) than those who do not use Facebook. Besides, agreeableness is not related to overall Facebook use (Ross et al., 2009; Moore and McElroy, 2012), but those high in agreeableness are especially likely to use Facebook as a tool for communicating with others (Moore and McElroy, 2012; Seidman, 2013). Ross et al. (2009) revealed that the direction of the relationship between neuroticism and Facebook use depends on the actions taken on that site: low neuroticism is associated with a preference for uploading images, and high neuroticism is related to the preference for writing on the site's general forum. Seidman (2013) found associations between the Big Five traits (Five-Factor Model of personality) and various motives for using Facebook—for example, extraversion and openness turned out to be related to communication, agreeableness to searching for information and expression of the actual self (positive associations), and conscientiousness to communication and expression of the ideal self (negative associations). As it turned out, high levels of agreeableness and neuroticism were predictors of the need for belonging. In contrast, a high level of neuroticism, and a low level of conscientiousness were predictors of the need for self-presentation (Seidman, 2013). The need for self-presentation is satisfied by the disclosure of information about oneself, posting pictures, and creating one's image on Facebook.

**Figure 1:**
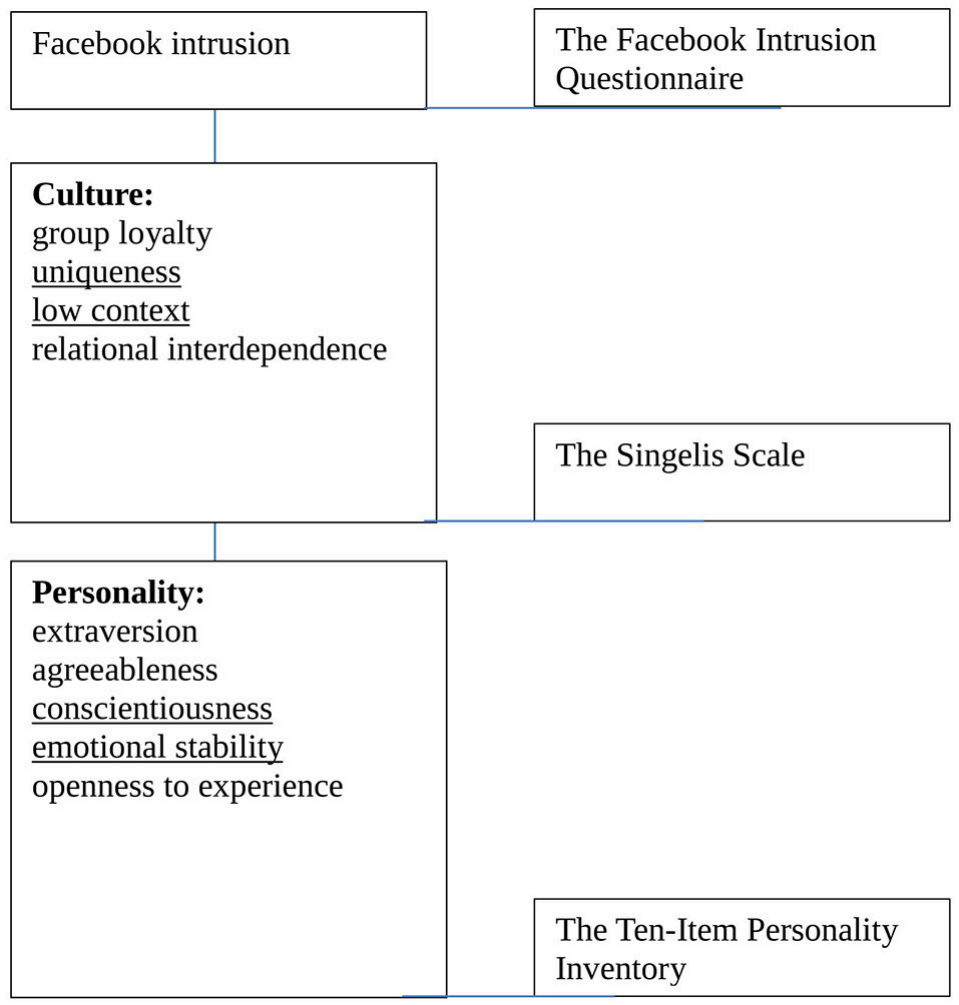
**The cultural and personality determinants of Facebook intrusion and instruments used to measure them**.

Our previous analysis revealed that the level of Facebook intrusion differs depending on the country of residence (Błachnio et al., 2016); therefore, looking for predictors of this phenomenon seems to be legitimate. The aim of the paper is to expand knowledge on the cultural and personality determinants of Facebook intrusion (see Figure 1). Based on the above overview of research the four factors related to culture (group loyalty, uniqueness, low context, and relational interdependence) as well as the five dimensions of personality (extraversion, agreeableness, conscientiousness, emotional stability, and openness to experience) can be expected to be associated with Facebook intrusion.

## Material and methods

### Participants

A total of 2628 Facebook users took part in the study conducted across eight countries, namely: China (*n* = 388), Greece (*n* = 253), Israel (*n* = 311), Italy (*n* = 317), Poland (*n* = 453), Romania (*n* = 273), Turkey (*n* = 395), and the USA (*n* = 238). In the whole sample, the mean age of the participants was 26.06 (*SD* = 10.71). As regards country-specific samples, the mean age was *M* = 19.07 (*SD* = 1.34) in China, *M* = 27.05 (*SD* = 9.05) in Greece, *M* = 32.14 (*SD* = 13.70) in Israel, *M* = 24.66 (*SD* = 6.52), in Italy, *M* = 35.01 (*SD* = 13.74) in Poland, *M* = 20.48 (*SD* = 1.83) in Romania, *M* = 23.56 (*SD* = 5.99) in Turkey, and *M* = 23.65 (*SD* = 10.96) in the USA. In the whole sample, 54% of the participants were women. The percentages of women in country-specific samples were as follows: 63% for China, 40% for Greece, 81% for Israel, 67% for Italy, 52% for Poland, 46% for Romania, 69% for Turkey, and 72% for the USA.

### Procedure

The participants fulfilled one criterion: they were Facebook users. We used the snowball sampling procedure to recruit participants. A link to online questionnaires was sent to Facebook users, who were informed about the anonymity of the research. The participants received no remuneration for taking part in our research project. None of them was excluded from the study. Some results of the survey, unrelated to the objective of the current study, have been presented elsewhere (Błachnio et al., 2016). It should be explained that Facebook is unavailable in some regions in China. The Chinese participants were asked not about Facebook use but about their use of “SNS.” Because the cross-cultural equivalence of the instrument measuring Facebook intrusion was acceptable for China (see below), we decided to add the data from the Chinese sample. Also worth noting is a study in which the Internet Addiction Test was adapted to the Facebook context and yielded reliable results (Dantlgraber et al., 2016). The study was approved by the ethics committee.

### Measures

The participants from eight countries completed an online survey measuring Facebook intrusion, cultural dimensions, and personality. All the questionnaires used had been previously published in English and were therefore translated into the native language of each country surveyed.

*The Facebook Intrusion Questionnaire*, developed by Elphinston and Noller (2011), is based on behavioral addiction components and on a scale measuring phone involvement. It consists of eight items (e.g., *I have been unable to reduce my Facebook use*) measuring the relations between the tendency to Facebook involvement and eight aspects of behavioral addiction, namely: cognitive salience, behavioral salience, interpersonal conflict, conflict with other activities, euphoria, loss of control, withdrawal, as well as relapse and reinstatement. The items are rated on a 7-point Likert scale from 1 (*strongly disagree*) to 7 (*strongly agree*). Chinese participants were asked about their use of “SNS” in each item instead.

To assess the factor structure equivalence of Facebook intrusion, we compared the results of factor analysis performed on the whole aggregated sample (all countries, *N* = 2628) with the solutions obtained in each of the eight countries (as in Sircova et al., 2014). As Facebook intrusion is a unidimensional measure, to extract a single general factor, we applied principal components analysis and extracted the first unrotated factor. The solution for the whole aggregated sample is presented in Table 1. Factor loadings for each person were saved using the regression method. This analysis was done first on the whole sample, and then in each country separately. The procedure yielded two factor loadings for each person—one relating to the pooled solution, and the second one to the country. We compared the two sets of factor loadings using Tucker's phi (Table 2).

**Table 1:** **Factor Loadings on the General Factor (Pooled Sample) for Facebook Intrusion Questionnaire**.

**Items**	**Loadings**
4. I interrupt whatever else I am doing when I feel the need to access Facebook (conflict with other activities)	0.79
1. I often think about Facebook when I am not using it (cognitive salience)	0.78
7. The thought of not being able to access Facebook makes me feel distressed (withdrawal)	0.77
8. I have been unable to reduce my Facebook use (relapse and reinstatement)	0.77
6. I lose track of how much I am using Facebook (loss of control)	0.68
5. I feel connected to others when I use Facebook (euphoria)	0.66
3. Arguments have arisen with others because of my Facebook use (interpersonal conflict)	0.64
2. I often use Facebook for no particular reason (behavioral salience)	0.50

**Table 2:** **Tucker's phi coefficients comparing the factor structure in each country to the pooled solution**.

**Country**	**Tucker's phi**
Poland	0.979
Israel	0.983
China	0.800
Romania	0.993
Italy	0.997
USA	0.988
Turkey	0.963
Greece	0.999

The analyses were done using SPSS 21 package. Tucker's phi coefficients were calculated by means of an Excel spreadsheet containing the formula.

The eigenvalue of the general factor was 3.99, and the percentage of variance explained was 49.82.

By all conventions, the Tucker's phi indicated (see Table 2) a good cross-cultural equivalence of Facebook intrusion (Lorenzo-Seva and Berge, 2006), but the results of the Chinese sample showed somewhat lower congruence, although acceptable at the lowest threshold.

*The Singelis Scale—*the shortened, 13-item version of Singelis (1994) scale—was used to measure Independent—Interdependent self-construal (Fernández et al., 2005). An example item is *I act the same way no matter who I am with.* The scale has four factors: group loyalty, uniqueness, low context, and relational interdependence. The items are rated on a 4-point Likert scale from 1 = *totally disagree* to 4 = *totally agree*.

*The Ten-Item Personality Inventory* (Gosling et al., 2003) measuring the Big Five dimensions: extraversion, agreeableness, conscientiousness, emotional stability, and openness to experience. It consists of 10 items, 2 items for each of the 5 dimensions. The items are characteristics related to each dimension, for instance extraverted, enthusiastic; reserved, quiet. The items are rated on a 7-point Likert scale from 1 = *strongly disagree* to 7 = *strongly agree*.

## Results

The correlations of Facebook intrusion with cultural variables (group loyalty, uniqueness, low context, and relational interdependence) and personality variables (extraversion, agreeableness, conscientiousness, emotional stability, and openness to experience) are presented in the Table 3.

**Table 3:** **Correlations of Facebook intrusion with cultural and personality variables**.

	**China**	**Greece**	**Israel**	**Italy**	**Poland**	**Romania**	**Turkey**	**USA**
Group loyalty	0.03	−0.12	0.16^***^	0.19^***^	0.22^***^	−0.09	0.06	0.09
Uniqueness	0.04	−0.10	0.06	0.05	−0.01	−0.13	0.02	−0.08
Low context	0.11	−0.36^***^	0.01	−0.15^**^	−0.10^*^	0.08	−0.07	−0.02
Relational interdependence	−0.04	−0.35^***^	0.01	0.08	−0.11^*^	0.01	0.03	0.23^***^
Extraversion	0.09	0.02	0.01	0.10	−0.05	−0.03	−0.17^***^	−0.12
Agreeableness	−0.05	−0.24^***^	−0.07	−0.11*	−0.21^***^	0.06	−0.13^**^	−0.06
Conscientiousness	−0.11^*^	−0.35^***^	−0.08	−0.27^***^	−0.33^***^	−0.03	−0.22^***^	−0.04
Emotional stability	−0.24^***^	−0.23^***^	−0.20^***^	−0.33^***^	−0.19^***^	−0.16^**^	−0.21^***^	−0.27^***^
Openness to experience	−0.09	−0.36^***^	0.04	−0.03	−0.20^***^	0.06	−0.16^***^	−0.18^**^

* p < 0.05;

** p < 0.01;

*** p < 0.001.

Analyzing the country variables and Facebook intrusion, we observed some interesting results. Group loyalty is positively related to Facebook intrusion in Israel, Italy, and Poland. Low context is negatively related to Facebook intrusion in Greece, Italy, and Poland. Relational independence is negatively connected with Facebook intrusion in Greece and Poland as well as positively in the USA.

As regards the relations between Facebook intrusion and personality, we found that emotional stability was negatively related to Facebook intrusion in all of the countries included in the study. Conscientiousness is negatively associated with Facebook intrusion in China, Greece, Italy, Poland, and Turkey. Agreeableness is negatively associated with Facebook intrusion in Greece, Italy, Poland, and Turkey. Openness to experience is negatively related to Facebook intrusion in Greece, Poland, Turkey, and the USA. Extraversion is negatively related to Facebook intrusion in Turkey.

### Multilevel analysis

To examine the role of Facebook intrusion in terms of the set of individual variables while controlling for and taking into account the variables related to Country (different cultures), we performed multilevel (hierarchical) analysis with Country as the variable in which the individual ones were nested. We used HLM 7 software (Raudenbush et al., 2011) to perform the multilevel analyses.

First, a null mode was created, in which the only predictor of the Facebook intrusion was Country. We used it to calculate the intra-class coefficient (ICC), which may be used to test whether multilevel testing is needed (a nonsignificant ICC would suggest that it is not). The ICC in the null model was 0.034 (*chi-square* = 58.21, *df* = 7, *p* < 0.001). The significant ICC suggested that multilevel modeling was needed, although it was rather low and perhaps significant due to the large sample.

Next, we tested a multilevel model, entering all variables as random. The intercept of the equation of personality variables (extraversion, agreeableness, conscientiousness, emotional stability, and openness to experience) was significantly associated with the random effect of Country and with the random effect of the variables related to Country: group loyalty, uniqueness, low context, and relational interdependence. Any significant effect of these variables would suggest that the mean score of the dependent variable is affected by them. In sum, the model was a two-level one. All variables were grand-mean centered before being entered into the model. The results are summarized in Table 4.

**Table 4:** **Results of two-level hierarchical modeling predicting Facebook intrusion from country and personality variables**.

**Fixed effects**	**B**	**SE**	***T***	***df***	***p***
**COUNTRY**					
Intercept	23.190	0.538	43.133	3	<0.001
Group loyalty	−2.463	1.103	−2.232	3	0.112
Uniqueness	−4.373	0.959	−4.560	3	**0.020**
Low context	4.386	1.106	3.966	3	**0.029**
Relational interdependence	0.578	0.712	0.811	3	0.477
Extraversion	0.359	0.230	1.564	7	0.162
Agreeableness	−0.139	0.262	−0.531	7	0.612
Conscientiousness	−1.001	0.414	−2.419	7	**0.046**
Emotional stability	−1.023	0.293	−3.489	7	**0.010**
Openness to experience	−0.549	0.456	−1.205	7	0.267

All significant results are marked in bold for clarity.

**Figure 2:**
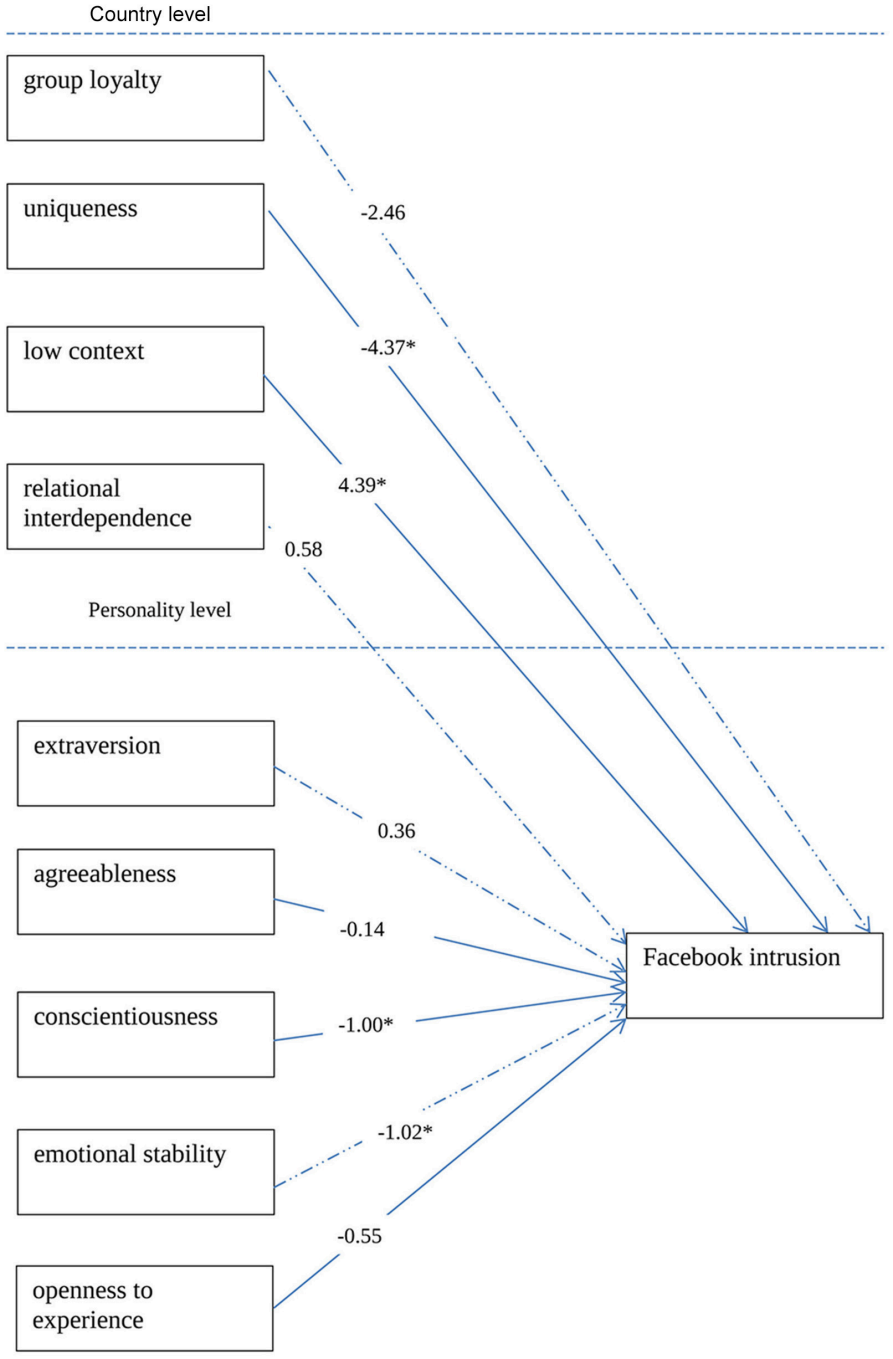
**Model explaining Facebook intrusion, the unstandardized data were used**.

The multilevel analyses including data from eight countries shows that some of the country and personality variables are predictors of Facebook intrusion (see Figure 2). As we can see in the Table 4, at the country level, uniqueness predicted Facebook intrusion negatively (*B* = −4.37, *p* = 0.02) and low context predicted it positively (*B* = 4.39, *p* = 0.03). As regards the personality level, both conscientiousness (*B* = −1.02, *p* = 0.04) and emotional stability (*B* = −1.02, *p* = 0.01) negatively predicted Facebook intrusion.

## Discussion

The aim of the paper was to examine the cultural and personality determinants of Facebook intrusion. Four factors related to country/culture (group loyalty, uniqueness, low context, and relational interdependence) as well as five dimensions of personality (extraversion, agreeableness, conscientiousness, emotional stability, and openness to experience) were taken into consideration. Different relations between country as well as personality variables and Facebook intrusion may indicate that those relations are not universal. It seems reasonable to conclude that there are cultural differences in the explanation of Facebook intrusion. We found that two cultural variables and two dimensions of personality were significant predictors of Facebook intrusion. On the level of culture, low uniqueness, and high context predict Facebook intrusion.

According to the present findings, there are some differences in the relations between personality variables and Facebook intrusion across the countries. Two variables were significant in all of the countries—namely, emotional stability and conscientiousness, which were negatively linked with Facebook intrusion in each country. People with low emotional stability are probably more often involved in Facebook in general. It can be assumed that those Facebook users who scored higher on emotional stability have less negative emotions and are less inclined to engage in problematic Facebook use. This result is in line with other research (e.g., Zamani et al., 2011; Kuss et al., 2014; Błachnio and Przepiorka, 2016), showing a positive relationship between emotional stability and Internet and Facebook addiction. As shown in previous studies, this pattern of relations was the same across different cultures and countries, for instance in Poland, Iran, the Netherlands, and UK. Similarly, other studies also found a link between conscientiousness and Internet or Facebook addiction (e.g., Stavropoulos et al., 2015; Błachnio and Przepiorka, 2016).

We found some differences in the relations between country variables and Facebook intrusion. In a similar vein, other research also provided evidence that culture shapes Facebook use (e.g., Abbas and Mesch, 2015). It showed that cultural values are associated with how people use Facebook. The present study gave insight into some cross-cultural differences in Facebook use. On the cultural level, people lacking a sense of uniqueness from others (i.e., low score on uniqueness) and people who prefer directness in communication (i.e., high score on low context) scored higher on Facebook intrusion. It seems that people who feel part of a group rather than as unique individuals more often become involved in Facebook. Probably this social network gives them an opportunity to be part of a community that highlights their identity. To support this assumption, we can refer to the basic motives of Facebook use. For instance, Nadkarni and Hofmann (2012) mentioned two basic social needs behind Facebook use. One of them is the need to belong, connected with affiliation with others and a desire for social acceptance. Facebook is a tool to boost social capital and to sustain the existing relationships or to build new ones (e.g., Ellison et al., 2007). Moreover, people who prefer direct, simple, and clear messages in communication are more often involved in Facebook. Facebook—and Internet in general—is a place where direct and explicit messages are required in communication (Postmes and Spears, 2013).

We should remember that the results demonstrate rather low ICC of the null model, which may suggest that the effects obtained are cross-culturally universal. Our findings may help to understand the pattern of Facebook intrusion. The results of the present study may be useful in formulating recommendations for the prevention of Facebook addiction across different cultures. Of the personality traits, it is emotional stability and conscientiousness that play a central role in Facebook intrusion. On the cultural level, low context and uniqueness should be considered in diagnosing and explaining the development of Facebook intrusion. Some previous studies suggested significant relationships between Facebook users' motivation and cultural dimensions such as masculinity or long-term orientation. What is more, the cultural value of motivation and the attitude toward SNS were taken into consideration in a study (Al Omoush et al., 2012). Cho (2010) indicated that people from different cultures differ in terms of Facebook usage motivation; for instance, members of collectivistic cultures focus on self-presentation more strongly than users from individualistic cultures.

On the level of personality, Facebook intrusion was predicted by low levels of both conscientiousness and emotional stability. On the personal level, easy-going and disorderly people as well as neurotic individuals often have the problem of excessive Facebook use. In a previous study, Wang et al. (2015) showed that neuroticism is associated with networking addiction among Chinese adolescents. Andreassen et al. (2013) pointed out that conscientiousness is associated negatively and neuroticism positively with Facebook addiction in Norwegians students. Similar results were obtained for Internet use among Turkish university students (Durak and Senol-Durak, 2014). However, Skues et al. (2012) indicated that there are not associations between extraversion, neuroticism, and Facebook use.

On the one hand, we obtained interesting results on quite a large sample; on the other, there are some limitations that should be mentioned. The first limitation of the study is the rather low ICC of the null model, which means the influence of cultural factors on Facebook intrusion is ambiguous. One of the limitations is the sampling frame. In particular, the participants' age varied in each country, although most of them were college students. On the other hand, the primary criterion was Facebook usage, which concerns all age groups. It should also be noted that the nature of the sampling method may have influenced the pattern of responses and the overall levels of activity, although the researchers have no reason to believe that the source of the sample biased the results. Creating a sampling frame adequately representing non-college users of Facebook has been a challenge and needs further work. Another exploratory study is needed in which more cultural variables should be taken into consideration.

Despite the above limitations, the study makes it possible to systematize knowledge on Facebook use and to create a model explaining the relationship between the personality and cultural characteristics and motives of Facebook use. In almost all cultures the popularity of SNS usage has been increasing every year, and knowledge about the determinants of Facebook use is of great importance.

To sum up, cultural and personality variables can predict Facebook addiction. As regards culture, uniqueness is negatively and low context positively related to Facebook intrusion, the relationship being significant in both cases. As regards the personality dimensions, conscientiousness and emotional stability are both significant negative predictors of Facebook intrusion.

## Author contributions

AB: preparing the manuscript, collecting the data in Poland. AP: preparing the mauscript, collecting the data in Poland. The rest of the Authors: collecting the data in their own countries, making alterations to the last version of the manuscript.

## Funding

This research was supported by a Grant from the NCN No.2014/15/B/HS6/03129. AP was supported by the Foundation for Polish Science (FNP) (FNP, START88.2015-W).

### Conflict of interest statement

The authors declare that the research was conducted in the absence of any commercial or financial relationships that could be construed as a potential conflict of interest.

## Footnotes

^1^http://newsroom.fb.com/company-info/
